# Lower socioeconomic status and the acceleration of aging: An outcome-wide analysis

**DOI:** 10.1073/pnas.1915741117

**Published:** 2020-06-15

**Authors:** Andrew Steptoe, Paola Zaninotto

**Affiliations:** ^a^Department of Epidemiology and Public Health, University College London, London WC1E 6BT, United Kingdom

**Keywords:** social disparities, aging, cognition, physical capability, social function

## Abstract

Aging involves decline in a range of functional abilities and phenotypes, many of which are also associated with socioeconomic status (SES). Here we assessed whether lower SES is a determinant of the rate of decline over 8 y in six domains—physical capability, sensory function, physiological function, cognitive performance, emotional well-being, and social function—in a sample of 5,018 men and women aged 64.44 (SD 8.49) y on average at baseline. Wealth was used as the marker of SES, and all analyses controlled for age, gender, ethnicity, educational attainment, and long-term health conditions. Lower SES was associated with greater adverse changes in physical capability (grip strength, gait speed, and physical activity), sensory function (sight impairment), physiological function (plasma fibrinogen concentration and lung function), cognitive performance (memory, executive function, and processing speed), emotional well-being (enjoyment of life and depressive symptoms), and social function (organizational membership, number of close friends, volunteering, and cultural engagement). Effects were maintained when controlling statistically for other factors such as smoking, marital/partnership status, and self-rated health and were also present when analyses were limited to participants aged ≤75 y. We conclude that lower SES is related to accelerated aging across a broad range of functional abilities and phenotypes independently of the presence of health conditions and that social circumstances impinge on multiple aspects of aging.

## Significance

Lower socioeconomic status (SES) is a determinant of many of the health problems that emerge at older ages. The extent to which lower SES is associated with faster decline in age-related functions and phenotypes independently of health conditions is less clear. This study demonstrates that lower SES (defined by wealth) is related to accelerated decline over 6 to 8 y in 16 outcomes from physical, sensory, physiological, cognitive, emotional, and social domains, independently of diagnosed health conditions, self-rated health, education, and other factors. It provides evidence for the pervasive role of social circumstances on core aging processes and suggests that less affluent sectors of society age more rapidly than more privileged groups.

Socioeconomic status (SES) is a major determinant of health, with people of lower SES being at increased risk of premature mortality; the development of serious conditions such as coronary heart disease, diabetes, and depression; and other health outcomes at older ages including disability and dementia (1–5). These associations are underpinned by biological processes such as chronic allostatic load and sustained inflammation and by lifestyle factors including smoking and sedentary behavior (6–9). A range of SES markers has been related to health at older ages, including indicators of childhood SES, education, occupational status, income, wealth, and area-based measures of deprivation.

It is less clear whether SES is a factor in aging processes independently of the development of diagnosed illnesses. Aging involves phenotypic and functional changes as well as molecular and cellular processes (10). These include declining cognition and sensory function, reduced physical capability, changes in physiological and biological function, and reductions in social engagement and activity, none of which are illnesses in themselves. There is good evidence that SES is associated cross-sectionally with impairment of these processes, with lower SES being related to poorer cognition (11), impairment in sight and hearing (12), slower walking speed and lower muscle strength (13, 14), poor physiological function and greater inflammation (15, 16), lower psychological well-being (17), and reduced social/cultural engagement and prosocial behavior (18, 19). Longitudinal evidence for SES being associated with change over time is more limited and inconsistent, with some studies showing accelerated decline among lower SES individuals (20, 21) and others showing slower decline (22, 23) or no differences in change across SES categories (24, 25).

In this study, we therefore assessed associations in a single dataset between SES and rate of functional and phenotypic aging in a representative sample of older men and women. We took an outcome-wide approach (26), assessing function across six domains: physical capability, sensory processes, physiological function and inflammation, cognitive function, emotional well-being, and social function. We hypothesized that lower SES would be associated cross-sectionally with impaired function and longitudinally with accelerated decline over an 8-y period independently of diagnosed illnesses. We indexed SES using wealth, controlling statistically for educational attainment, since accumulation of wealth appears to be a more robust indicator of socioeconomic resources than occupational status or income at older ages (27, 28).

## Results

The study involved 5,018 participants in the English Longitudinal Study of Aging (ELSA) aged 52 and over (mean 64.44, SD 8.49) assessed in 2004 (baseline) and then 8 y later in 2012. Wealth was divided into quartiles for the purposes of analysis. All analyses included age, age squared (to account for nonlinear age associations), gender, ethnicity, educational attainment, childhood SES (based on paternal or primary caregiver’s occupation when the participant was aged 14), and number of long-term conditions (arthritis, asthma, cancer, coronary heart disease, dementia, diabetes, heart failure, Parkinson’s disease, and stroke) as covariates. As can be seen in *SI Appendix*, Table S1, lower SES participants were slightly older on average; were more likely to be female and nonwhite (although the overall proportion of ethnic minorities was small); and had fewer educational qualifications, lower childhood SES, and more long-term conditions. All analyses were weighted using inverse probability weights to ensure national representation and to take account of differential nonresponse at follow-up (see *SI Appendix* for details). The levels of the 19 outcome variables at baseline and follow-up are summarized in *SI Appendix*, Table S2; the numbers in each analysis varied because of missing data. Bonferroni corrections were applied within each domain to take account of multiple comparisons.

The cross-sectional associations between SES and outcomes are detailed in Table 1. The four measures of physical capability were all related to SES in a graded fashion, with lower hand grip strength, slower gait speed over a standard distance of 8 ft, reduced ability to reach criteria in standing up repeatedly from a chair, and less self-reported physical activity among lower SES participants. Self-reported sensory function was also inversely associated with SES, with lower SES participants more likely to rate their sight only fair or poor, rather than good, very good, or excellent. The association with self-reported hearing was not significant after Bonferroni correction. In the physiological function domain, lower SES was related to higher levels of two markers of inflammation: high-sensitivity plasma C-reactive protein and fibrinogen. There was also a gradient in lung function, with lower forced expiratory volume over 1 s (FEV_1_) and forced vital capacity (FVC) in less affluent participants.

**Table 1.** Cross-sectional associations of SES with outcomes

Outcome	Wealth quartile				*P*
	Mean (SE) or odds ratio (95% CI)				
	1 (highest)	2	3	4 (lowest)	
Physical capability					
Grip strength (kg)	31.68 (0.185)	31.21 (0.186)	30.41 (0.197)	29.66 (0.229)	<0.001
Gait speed (m/s)	0.990 (0.008)	0.932 (0.0098)	0.892 (0.009)	0.832 (0.010)	<0.001
Chair stand failure: OR	1 (ref)	1.12 (0.85–1.48)	1. 55 (1.18–2.03)	2.43 (1.85–3.20)	<0.001
Physical activity index	2.48 (0.031)	2.38 (0.032)	2.24 (0.033)	1.97 (0.039)	<0.001
Sensory function					
Sight (fair/poor): OR	1 (ref)	1.20 (0.89–1.61)	1.56 (1.17–2.09)	2.33 (1.74–3.13)	<0.001
Hearing (fair/poor): OR	1 (ref)	1.02 (0.82–1.26)	1.30 (1.04–1.61)	1.22 (0.96–1.54)	0.049*
Markers of physiological function					
C-reaction protein (mg/L)	2.48 (0.094)	2.82 (0.096)	3.23 (0.102)	3.27 (0.119)	<0.001
Fibrinogen (g/L)	3.10 (0.021)	3.19 (0.021)	3.22 (0.022)	3.21 (0.026)	0.001
FEV % predicted	98.34 (0.661)	95.63 (0.668)	92.91 (0.714)	92.30 (0.841)	<0.001
FVC (L)	3.41 (0.022)	3.32 (0.022)	3.21 (0.024)	3.14 (0.028)	<0.001
Cognitive function					
Memory (n items)	10.96 (0.078)	10.69 (0.078)	10.54 (0.0823	10.13 (0.096)	<0.001
Verbal fluency (n items)	21.29 (0.151)	20.68 (0.151)	20.47 (0.158)	19.91 (0.180)	<0.001
Processing speed (n items)	306.0 (2.29)	304.4 (2.30)	298.7 (2.41)	300.6 (2.77)	0.066
Emotional well-being					
Enjoyment of life	10.49 (0.045)	10.34 (0.046)	10.16 (0.049)	9.79 (0.057)	<0.001
Depressive symptoms: OR	1 (ref)	1.26 (1.02–1.58)	1.58 (1.26–1.98)	2.14 (1.69–2.70)	<0.001
Social function					
Organizational membership (n)	1.90 (0.040)	1.62 (0.039)	1.48 (0.041)	1.27 (0.048)	<0.001
Close friends (n)	3.77 (0.077)	3.56 (0.073)	3.49 (0.078)	3.21 (0.094)	<0.001
Volunteer: OR	1 (ref)	0.76 (0.63–0.91)	0.60 (0.49–0.74)	0.51 (0.40–0.64)	<0.001
Cultural engagement: OR	1 (ref)	0.67 (0.57–0.79)	0.62 (0.51–0.74)	0.44 (0.35–0.55)	<0.001

All analyses are adjusted for baseline age, age^2^, gender, ethnicity, education, and number of long-term conditions. *P* is for trend across SES groups. OR, odds ratio; ref, reference category.

* Not significant after correction for multiple comparisons.

Three aspects of cognitive function were assessed: memory (a combination of immediate and delayed recall of word lists), executive function (a verbal fluency task), and processing speed on a letter cancellation task (29). There were significant cross-sectional SES gradients in memory and executive function but not processing speed, with poorer memory and executive function in lower SES categories. We measured two aspects of emotional well-being. Positive affective well-being was assessed with a four-item measure of enjoyment of life that has previously been associated with health outcomes (30), while depressive symptoms were indexed by the eight-item Center for Epidemiologic Studies Depression scale (CESD) (31). Both showed SES gradients, with lower positive well-being and an increased prevalence of depressive symptoms in lower SES groups. In the social domain, we included four indicators of social function and prosocial behavior: participation in organizations such as social clubs, church groups, and resident organizations; number of close friends; volunteering on a monthly basis or more frequently; and cultural engagement (going to museums, concerts, or theater). In all cases, there was a significant social gradient, with lower SES individuals being less likely to participate.

**Fig. 1.**
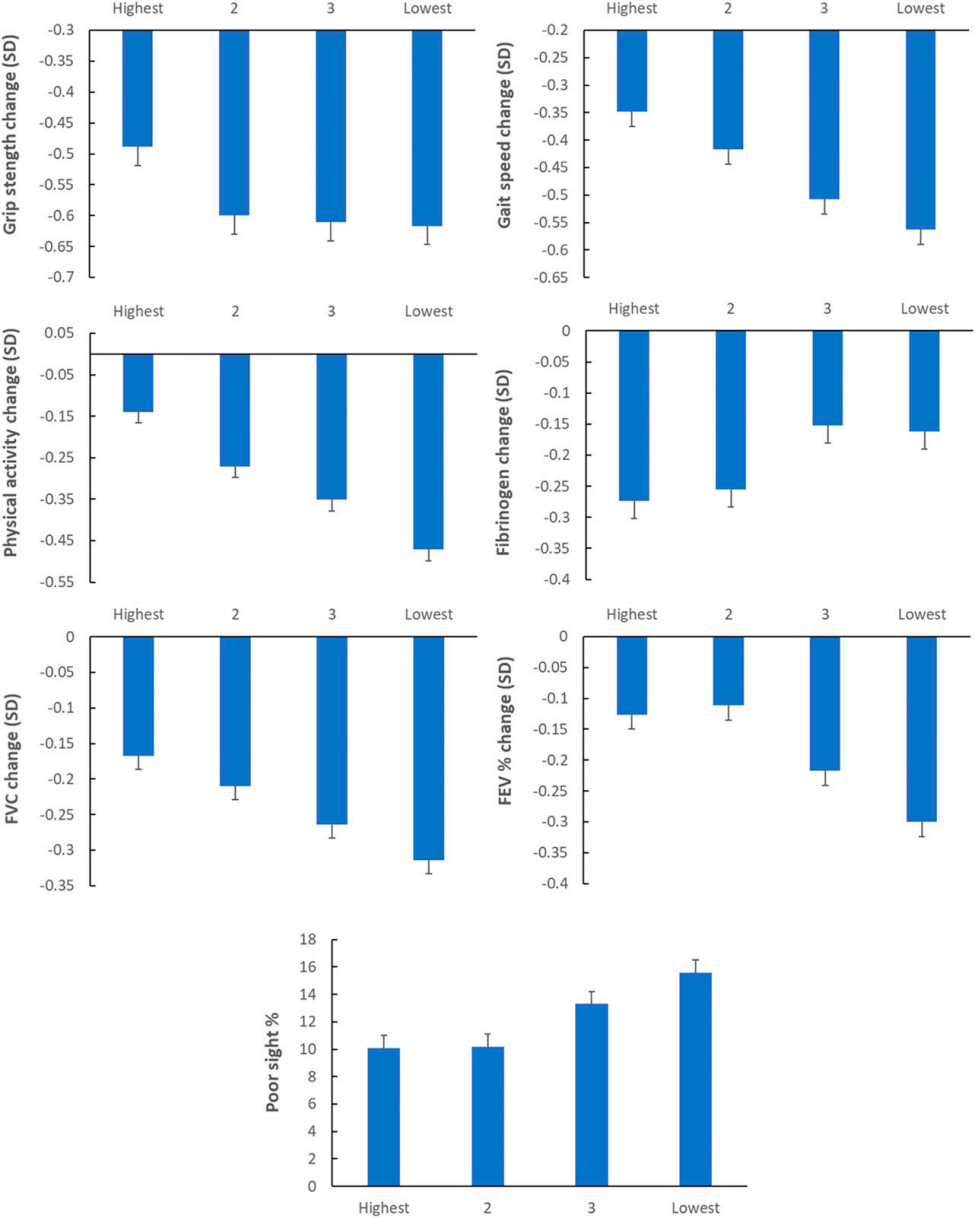
Changes over 8 y in physical capability (grip strength, gait speed, and physical activity), physiological function (fibrinogen concentration, FEV, and FVC), and sensory function (incident poor sight) in relation to SES categorized into quartiles of wealth (highest to lowest). Values are adjusted for age, age^2^, gender, education, childhood SES, number of long-term conditions, and baseline levels of the outcome. Error bars are SEM.

**Fig. 2.**
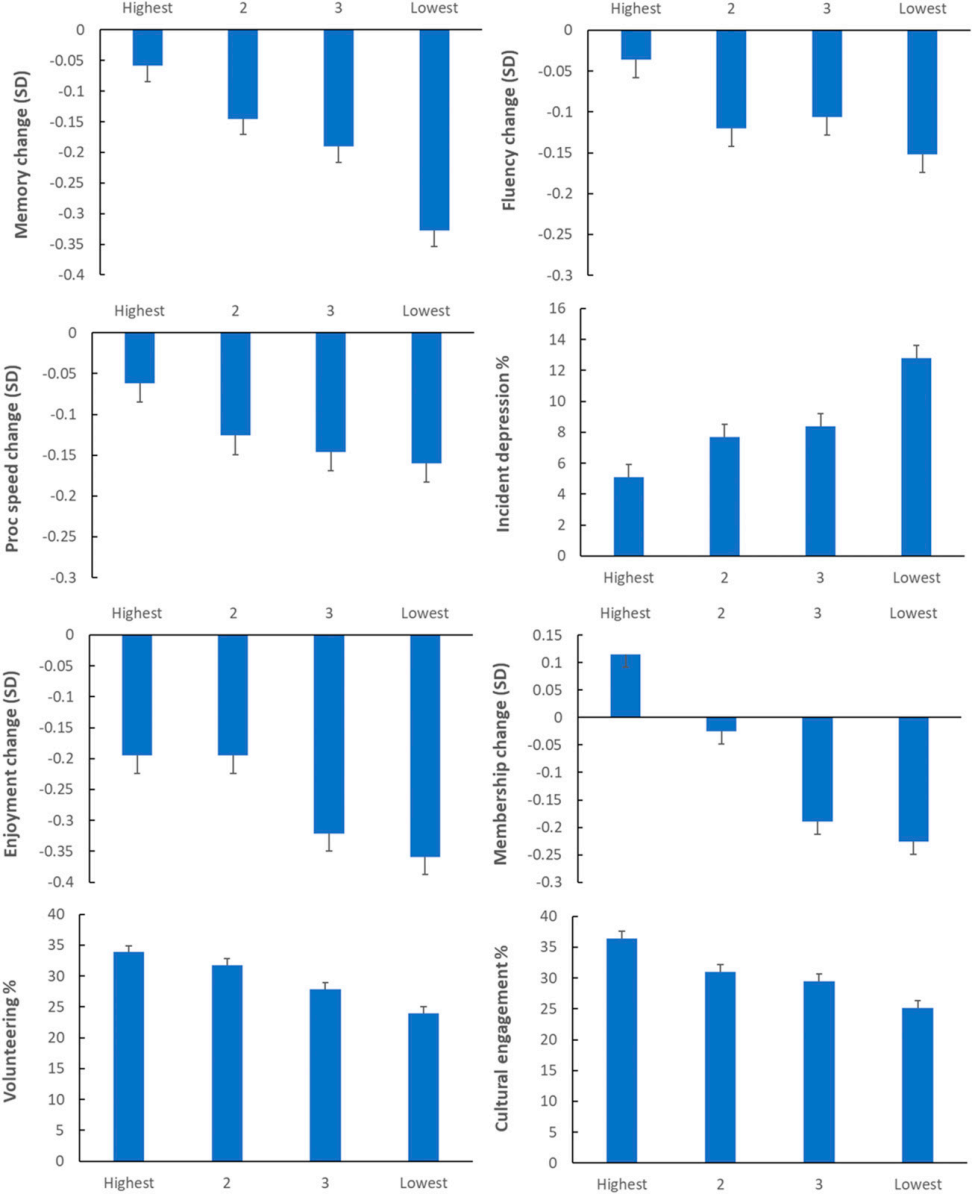
Changes over 8 y in cognitive function (memory, verbal fluency over 6 y, and processing speed over 6 y), emotional well-being (enjoyment of life and incident depressive symptoms), and social function (group membership, volunteering, and cultural engagement) in relation to SES categorized into quartiles of wealth (highest to lowest). Values are adjusted for age, gender, age^2^, education, childhood SES, number of long-term conditions, and baseline levels of the outcome (except for depression, volunteering, and cultural engagement). Error bars are SEM.

Longitudinal analyses investigated SES differences in deterioration of function over 8 y. Analyses adjusted for baseline levels of each function and the same covariates as in the cross-sectional analyses. Significant results are presented in Figs. 1 and 2, with full statistical details in *SI Appendix*, Table S3. Because the continuous outcomes were measured on a variety of metrics, we standardized scores into SD changes. In the physical capability domain, there were reductions in average grip strength, gait speed, and self-reported physical activity over time. These decreases were graded by SES, being greater in the less wealthy categories. Differences were substantial; for instance, the reduction in gait speed was 38% greater in the lowest than in the highest wealth category. Although a higher proportion of people in the lower SES groups failed the chair stand test, the linear trend across categories was not significant. In the sensory function domain, incident self-reported sight problems ranged from 10.1% in the highest to 15.6% in the lowest SES category. The SES gradient in incident hearing problems was not significant.

Three of the four markers of physiological function showed social graded impairments over the 8 y follow-up period. The concentrations of both inflammatory markers decreased on average, and the reductions in fibrinogen were greater in higher SES groups. Lung function declined in all groups, with the decreases in FEV_1_ and FVC being significantly greater in lower SES participants after controlling for covariates. As can be seen in Fig. 1, the fall in FEV_1_ in the lowest SES group was more than double that in the highest group.

Changes in memory were assessed over 8 y, but the changes in executive function and processing speed were measured over a 6-y period because these tests were not administered in wave 6 but in wave 5 (2010) of ELSA. As can be seen in Fig. 2, performance of all three cognitive tests deteriorated over time, with fewer words recalled in the memory test, reduced success in the verbal fluency test, and slower speed on the letter cancellation task. In all cases, the decline was socially graded, with little change in the highest SES group progressing to larger reductions in the lowest SES group. Changes in the two measures of emotional well-being were also socially graded, with greater reductions in enjoyment of life and more incident depressive symptoms in the lower SES categories. There were also significant SES differences in changes over time in three of the four social function measures. Membership of organizations tended to decline but was maintained at baseline levels in the highest SES group. The likelihood of volunteering and of engaging in cultural activities on a regular basis also varied across SES groups, being greater in more affluent categories.

We carried out a number of sensitivity analyses to explore different explanations of results. First, we repeated analyses after omitting baseline levels of the outcomes as covariates. The results (*SI Appendix*, Table S4) show no changes in the pattern of results for incident or binary outcomes but reduced statistical significance for the continuously distributed outcomes. Only the differences in changes in memory remained significant, indicating that the SES variations in decline were dependent on cross-sectional differences. Second, analyses were limited to people aged 75 and younger. The results in *SI Appendix*, Table S5, show a similar pattern as in the full analysis, with 12 of the 15 significant associations being unchanged. However, the SES differences in grip strength, sight impairment, verbal fluency, and processing speed were no longer robust, whereas the gradient in C-reactive protein concentration was significant. Third, we tested the possibility that differences in smoking rates underlie the pattern of results. Smoking is socially graded and is associated with the acceleration of cognitive decline, inflammation, and several other outcomes. Smoking rates were 8.3% in the highest wealth quartile, increasing to 10.3, 14.7, and 24.9% across the remaining quartiles. However, adding smoking as a covariate had a limited influence on longitudinal results, with three associations no longer being significant (grip strength, sight impairment, and verbal fluency) (*SI Appendix*, Table S6). Next, we considered whether differences in marital status were influential since marriage/partnership is more common among higher SES individuals and is related to several of the outcomes assessed here (32, 33). Repeating the analyses with baseline marital status as a covariate resulted in only one change (grip strength) to the significance of longitudinal associations. Finally, we conjectured that although the associations in Figs. 1 and 2 were independent of the presence of long-term health conditions, there might be health effects that were not detected by these measures. We therefore repeated the analyses adding self-rated health as a covariate. The associations between SES and grip strength and incident sight impairment were no longer significant, but there were no other differences from the main analyses.

## Discussion

Lower SES is associated with increased risk of many of the long-term health problems of aging, and declines in function and phenotypes could be consequences of SES gradients in illness. Our results indicate that lower SES is related to acceleration of a broad range of age-related impairments independently of diagnosed health conditions or self-rated health. It is striking that these associations were observed across six domains of physical, mental, and social function that typically deteriorate with age. Associations were independent of age, gender, ethnicity, educational attainment, and childhood SES as well as reported illness status. Although the overall magnitude of decline varied across outcomes, SES differences in change were similar, averaging 0.1 to 0.2 SDs between the highest and lowest SES groups. Sensitivity analyses indicated that the pattern of results could not be explained by the influence of smoking status, marital status, or additional indicators of health and were present in younger participants as well as the full sample.

The cross-sectional results summarized in Table 1 largely corroborate previous findings (11–15, 18) but provide little insight into the temporal relationships. Processes such as sensory impairment, inflammation, and low psychological well-being could potentially contribute to reduced SES as well as being exacerbated by lower SES. Most existing longitudinal studies of SES and age-related decline have focused on individual domains such as cognitive function or physical capability and have shown mixed relationships (11, 14, 21, 22). Work combining different outcomes has been carried out in the successful aging research literature, but findings have varied across domains of function and measures of SES (16, 34, 35). Our approach used a single indicator of SES (wealth), relating it to 19 outcomes in a single dataset, applying a uniform approach to analysis. This outcome-wide approach has been advocated for population studies because it provides information about the broad impact of specific exposures and avoids many of the methodological difficulties of exposure-wide studies (26). We selected wealth as a more precise indicator of contemporary socioeconomic resources at older ages than measures such as education and occupational status that are typically acquired in early life or midlife. Income can also be misleading since some older people with financial resources seek to limit their incomes in order to preserve their capital (28). All analyses also took childhood SES and educational attainment into account, indicating that the differences with wealth were not proxies for earlier life SES but represented accumulated socioeconomic resources at older ages.

The longitudinal analyses of continuously distributed variables were conducted with baseline levels of the outcomes as covariates in order to take account of the observed cross-sectional associations. Although it has been argued that adjustment for baseline may not be appropriate (36), this is a controversial issue because simple analyses of change scores may not provide unbiased estimates (37, 38), and the inclusion of baseline covariates has been advocated on statistical grounds, including improving efficiency, precision, and power and to avoid the pitfall of regression to the mean (39, 40). To explore and evaluate the differences in results we conducted sensitivity analyses excluding baseline covariates; findings for the six incident or binary outcomes were unchanged, but for the continuous outcomes the magnitude of several of the coefficients was substantially diminished, consequently affecting statistical significance. Previous studies have shown that changes in many capability outcomes are highly influenced by baseline values (14, 23, 41); therefore, we believe that a more appropriate approach in this setting is the use of baseline adjustment.

No single factor is likely to drive these associations between the extent of age-related decline and SES. Rather, multiple processes associated with SES disparities may contribute to different domains of function. For instance, greater wealth may provide a pathway toward more mentally stimulating environments and cultural resources that will impact on cognitive and social function; better access to green spaces and exercise facilities may enhance physical activity and help maintain physical capability; wealth may be directly related to eye care; and greater chronic life stress in less affluent groups may influence mental well-being and inflammation, while exposure to environmental pollution may promote faster decline in lung function among lower SES groups.

This study involved a large representative sample of older people in England and included a combination of self-reported and objective outcomes. The multifactorial nature of data in ELSA provided the opportunity to evaluate a broad range of outcomes and to explore several potential explanations of the findings. However, as with most longitudinal studies, there was marked attrition over the 8-y follow-up period. Part of this was because of deaths which were more common among lower SES participants. Of the original sample, 18.7% died before the 8 y follow-up, with death rates ranging from 29.9% in the lowest to 15.7% in the highest SES group. This survivorship bias might be expected to act against the hypotheses tested because the participants lost to the study would have been more vulnerable to functional decline, leaving a more robust lower SES group. We addressed this issue by inverse probability weighting to account for several factors associated with attrition. Unfortunately, there were some facets of age-related decline that could not be assessed, including changes in sleep patterns and nutrition. Other variables such as body mass index could have been analyzed but were excluded because the influence of aging on changes in adiposity independently of ill health is not clear.

Ferrucci et al. (10) have argued there is a hierarchical relationship between the molecular/cellular, phenotypic and functional levels in aging, with alterations at the basic biological level being buffered by compensatory resilience mechanisms that delay their impact on phenotypic processes, that in turn drive functional impairment. Whether lower SES impacts on biological and cellular aging processes is poorly understood; research to date has involved cross-sectional studies using education as a marker of early life SES, and inconsistent associations with telomere length and epigenetic aging have been reported (42–44). This study provides evidence for pervasive associations between lower SES and the acceleration of decline across a wide range of age-related processes at the functional and phenotypic levels. The observation that relationships were independent of reported health conditions and self-rated health suggests that SES impacts on important aging metrics directly. Such differences might persist even if social inequalities in long-term health conditions were reduced.

## Materials and Methods

ELSA is a nationally representative longitudinal panel study of English adults aged 50 and older (45). The study was approved through the National Research Ethics Service, and all participants provided informed consent. The baseline for these analyses was wave 2 (2004/2005) of data collection, and follow-up was in wave 6 (2012/2013). There were 8,402 participants with complete data on wealth (SES) and covariates at baseline, of whom 1,588 passed away, and 1,796 did not participate in wave 6, leaving 5,018 in the analytic sample. Wealth was measured with a detailed assessment of the participant’s financial, housing, and physical wealth (such as land, business wealth, and jewelry) but excluded pension wealth. The mean wealth in the four quartiles was £665,064, £247,413, £143,252, and £25,783, for the highest to lowest, respectively. The covariates in all analyses were age, gender, ethnicity (white or nonwhite), education divided into three levels of attainment, childhood SES, and the number of long-term health conditions present. The data used in these analyses are available from the UK Data Service (access GN 33368) at ukdataservice.ac.uk/.

Full details of the measures are provided in *SI Appendix*.

### Physical Capability.

Grip strength was assessed with a hand dynamometer; gait speed was measured with two 8-ft walking tests among respondents aged ≥ 60 y; the chair stand test involved repeated chair rises without using the arms, with age-dependent thresholds for success and failure; and physical activity was measured by self-report of vigorous, moderate, and light intensity activities, categorized into a five-point index from 1 indicating low to 5 indicating very active.

### Sensory Function.

Participants were asked to rate their eyesight and hearing (using spectacles and hearing aids if appropriate) as excellent, very good, good, fair, or poor.

### Markers of Physiological Function.

High-sensitivity C-reactive protein and fibrinogen concentrations were measured from blood samples obtained during home assessments by study nurses. FEV_1_ and FVC were assessed by spirometry, and FEV_1_ was expressed in terms of percent predicted values.

### Cognitive Function.

Memory was measured with a combination of immediate and delayed recall. Verbal fluency was assessed with the number of animals named in 1 min, while processing speed was monitored on a letter cancellation task. Verbal fluency and processing speed were not assessed in wave 6 but in wave 5 (2010) of ELSA, so the follow-up period was 6 rather than 8 y.

### Emotional Well-Being.

Enjoyment of life was measured with four items from the CASP questionnaire. Depressive symptoms were assessed with the eight-item CESD scale, using ≥4 as the threshold for significant depressive symptoms.

### Social Function.

Participants were asked about their membership of eight types of organization or club (e.g., tenant or resident group, church, and social clubs). They were also asked about the number of close friends they had; volunteering was indexed by whether or not the person volunteered at least once a month; and cultural engagement was categorized as going to art galleries, museums, theater, etc., at least every few months or not.

### Statistical Analysis.

Analysis of covariance was used to analyze continuously distributed outcomes with wealth (quartile) as the between-person factor and age, gender, ethnicity, and number of long-term conditions as covariates. *P* values for linear contrasts are presented. In the longitudinal analyses, difference scores between baseline and follow-up were analyzed, controlling for the same covariates plus the baseline level of the outcome measure. Details of the standardization of change scores are provided in *SI Appendix*. Results are presented as covariance-adjusted means and SE. Bonferroni corrections were applied to take account of multiple comparisons. Categorical outcomes were analyzed using logistic regression, and odds ratios (with 95% confidence intervals) adjusted for covariates are presented, with the highest wealth quartile as the reference group. Cross-sectional analyses were weighted for nonresponse to wave 2, while longitudinal analyses were weighted for participation in wave 6 of ELSA.

## Supplementary Material

Supplementary File
