# Supplementary material for: Lower socioeconomic status and the acceleration of aging: An outcome-wide analysis
Source: Proc Natl Acad Sci U S A. 2020 Jun 15;117(26):14911–7. doi: 10.1073/pnas.1915741117 (PMC7334539; doi:10.1073/pnas.1915741117)
Supplement: Supplementary File [file pnas.1915741117.sapp.pdf]

Lower Socioeconomic Status and the Acceleration of Aging: An outcome-wide analysis

Supporting Information

Data source

Data were analyzed from the English Longitudinal Study of Ageing (ELSA), a longitudinal panel study of men and women aged 50 and older living in England that started in 2002. The sample is assessed on a two yearly basis and biomarkers are collected every four years. The sample is periodically refreshed to ensure the full age range is maintained. Comparisons of sociodemographic characteristics with the national census show that the sample was representative of the English population. The questionnaires and general methods of data collection are detailed at www.elsa-project.ac.uk. The baseline for these analyses was wave 2 (2004), with follow-ups at waves 5 (2010) and 6 (2012). The study was approved at every wave through the National Ethics Service, and participants gave informed consent. Data are available from the UK Data Service (https://www.ukdataservice.ac.uk) and the Gateway to Global Aging (http://g2aging.org).

Details of measures

ELSA has three modes of data collection: computer-assisted personal interviews (CAPI) conduced by a trained interviewer in the person's home; self-completion (SC) questionnaires that are completed by participants in their own time and sent back to the research center; Nurse visits (NV) to the person's home for measurement of physiological function and collection of blood samples. Not all participants return the SC questionnaires or have NV, and some are excluded from blood sampling, as detailed in www.elsa-project.ac.uk. Inverse probably weights were applied to take account of differential response rates as detailed below. Descriptions of the measures indicate how they were collected.

Covariates

Age (CAPI) was modeled as a continuous variable. Age squared was included to take account of nonlinear aging effects.
Gender (CAPI) was divided into male and female, with men being the reference category in all analyses.
Education (CAPI). Educational attainment was measured as the person's highest educational qualification, divided into three categories: *Lower* (no qualifications), *Intermediate* (O levels and equivalent, indicating attainment of qualifications at the end of state-regulated schooling), and *Higher* (A level or equivalent up to university degree), The reference group in analyses was the no qualification category.
Childhood SES (CAPI) was based on the occupational status of the participant's father or guardian when the individual was aged 14. Occupations were classified into 3 categories: routine jobs (lower SES), intermediate, and professional / managerial (higher SES).
Long-term conditions (CAPI). The number of long-term health conditions (arthritis, asthma, cancer, coronary heart disease, dementia, diabetes, heart failure, Parkinson's disease, stroke) based on self-report of physician diagnosis was assessed in wave 2 (2004) for cross-sectional analyses, and in wave 6 (2012) for longitudinal analyses. Such reports have been shown to be reliable in comparison with medical records (1).
Additional covariates added in sensitivity analyses included smoking (current vs former or non-smoker), marital status (married/partnership), and self-rated health (excellent, very good, good, fair, or poor).

Outcomes

Physical capability
Grip strength (NV), a measure of upper body strength, was assessed using a Smedley's hand dynamometer. Respondents were asked to squeeze the dynamometer as hard as they could for 2 seconds, carrying out 3 trials with the dominant and 3 with the nondominant hand. The average maximum strength across both hands in Kg was analyzed as a continuous variable.
Gait speed (CAPI) is an objective test of physical function. It was assessed with two 8-foot walking tests from a standing start by respondents aged ≥ 60 years. The tests were carried out in the participants' homes under the supervision of a trained interviewer. Individuals who had health conditions or disabilities that prevented walking were not eligible for the test. Gait speed (in m/s) was computed.
Chair stands (NV) are part of the Short Physical Performance Battery (2). Participants were asked to stand up and sit down on a firm chair without using their arms as quickly as they could for five rises if they were aged 70 years or over, or up to ten rises if aged 69 years or under. Individuals who did not reach these numbers were classified as chair stand failures.
Physical activity (CAPI). Participants were asked the frequency with which they took part in sports or activities that were vigorous (e.g. jogging, cycling, aerobics), moderately energetic (e.g. gardening, walking at moderate pace) or mildly energetic (e.g. laundry, home repairs). The examples of activities for each question were those most commonly reported in previous UK-based population cohorts of this age. These were categorised according to the activity's metabolic equivalent (MET) score. These data were then used to compute a 5-point physical activity index where 0 (Sedentary) = no moderate and no vigorous activity; 1 = moderate activity once a week or 1–3 times a month and no vigorous activity; 2 = Moderate activity more than once a week and no vigorous activity, or vigorous activity 1–3 times a month and no moderate activity; 3 = Moderate activity once a week or more and vigorous activity once a week or 1–3 times a month, or vigorous activity once a week and moderate activity 1–3 times a month or never, or moderate activity 1–3 times a month and vigorous activity 1–3 times a month; 4 (Active) = vigorous activity more than once a week, with or without moderate activity.

Sensory function
Participants were asked to rate their eyesight (using spectacles if appropriate) and hearing (using hearing aids if appropriate) as excellent, very good, good, fair or poor. Ratings were subsequently categorized into fair/poor and compared with excellent, very good, or good.

Physiological function
During the nurse home visit, participants gave blood samples and had lung function measurements taken. High sensitivity C-reactive protein and fibrinogen concentrations were measured from blood samples obtained during home assessments. FEV_1 and FVC were assessed by spirometry, and FEV_1 was expressed in terms of % predicted values.

Cognitive function
Three aspects of cognitive function were assessed (3): memory (immediate and delayed recall), verbal fluency, and processing speed. In the memory test, participants were presented with a list of 10 words that were read out at the rate of one word for every 2 seconds. A total of four such lists were available, and these were randomly allocated by computer. After the presentation of words, participants were asked to recall as many words as they could (immediate recall). Participants were also asked to recall these words after an interval during which they completed other cognitive tests (delayed recall). The combined number of correctly recalled words was used as a measure of memory. Verbal fluency was used as a measure of executive function. Participants were asked to name as many animals as they could in 1 minute. Processing speed was measured using a letter cancellation task. Participants were given a page of random letters, and were asked to cross out as

many target letters (P and W) as quickly and accurately as possible within one minute. The processing speed score was the total number of letters searched, so higher scores indicate faster processing. Neither verbal fluency or processing speed were measured in wave 6 of ELSA, so the follow-up period for these variables was 6 rather than 8 years.

Emotional wellbeing
Positive affective wellbeing was assessed with 4 items from the CASP quality of life scale (4), as described previously (5). Each item ("I enjoy the things that I do"; "I enjoy being in the company of others"; "On balance, I look back on my life with a sense of happiness"; "I feel full of energy these days") was assessed on a four point scale. A total score was derived by adding responses from the four items.

Social variables
Organizations. Respondents were asked if they belonged to 8 types of organization, club or society: trade union or environmental group, tenant or resident group, church, charitable association, education, arts or music groups or evening classes, social clubs, sports clubs or gyms, or any other type of organization.
Number of close relationships was computed by asking respondents about the number of children, other family or friends with whom they have a close relationship. The maximum number in each category was censored at 10, so scores could range from 0 to 30. Close relationships were analyzed as a continuously distributed variable in cross-sectional and longitudinal analyses.
Volunteering was assessed as a measure of prosocial behavior. Participants were asked whether they carried out any volunteer work. Individuals who volunteered at least once per month were classified as volunteers.
Cultural engagement. Participants were asked how frequently they went to art galleries, museums, theatre, concerts or opera, with the response options *twice a month or more, about once a month, every few months, about once or twice a year, less than once a year,* or *never*. Individuals who attended at least every few months were categorized as culturally active. In longitudinal analyses, we assessed the proportion that was culturally active in 2016 that had not been active in 2012.

Standardization of change scores

Change scores in the longitudinal analyses of continuously distributed variables were calculated to provide a common metric across outcomes. The sex-specific standard deviation (SD) of each variable at baseline was computed, and both the baseline and follow-up values were divided by this number. Change scores were then calculated as the difference between these two standardized values.

Sample weighting in analyses

Inverse probably weights were applied to all analyses to take account of differential non-response and attrition rates. Separate weights were computed for CAPI, SC and NV variables, and also for variables dependent on blood collection. Sample weights for wave 2 (2004) were applied in the cross-sectional analyses, and for wave 6 (2012) or wave 5 (2010) for two cognitive measures in the longitudinal analyses. The details underlying the weights are detailed in the reports accompanying completion of these waves of data collection (6,7) and are available in the dataset curated in the UK Data Archive. Briefly, the factors taken into account include occupational status, limiting longstanding illness, region of the country, housing tenure (renting rather than owning), marital status, education, smoking, and age, with additional factors for SC, NV and blood weights. These weights ensure that the respondent age-sex distribution matches that of the most recent national census.

References

1. Simpson CF*, et al.* (2004) Agreement between self-report of disease diagnoses and medical record validation in disabled older women: factors that modify agreement. *J Am Geriatr Soc* 52(1):123-127.
2. Guralnik JM*, et al.* (2000) Lower extremity function and subsequent disability: consistency across studies, predictive models, and value of gait speed alone compared with the short physical performance battery. *J Geront. A, Biol Sci Med Sci* 55(4):M221-231.
3. Steel N, Huppert FA, McWilliams B, & Melzer D (2003) Physical and cognitive function. *Health, Wealth and Lifestyles of the Older Population in England*, eds Marmot M, Banks J, Blundell R, Lessof C, & Nazroo J (Institute for Fiscal Studies, London), pp 249-300.
4. Hyde M, Wiggins RD, Higgs P, & Blane DB (2003) A measure of quality of life in early old age: the theory, development and properties of a needs satisfaction model (CASP-19). *Aging Ment Health* 7(3):186-194.
5. Zaninotto P, Wardle J, & Steptoe A (2016) Sustained enjoyment of life and mortality at older ages: analysis of the English Longitudinal Study of Ageing. *BMJ* 355:i6267.
6. Bridges S, Hussey D, Blake M, & Philo D (2014) Methodology. *The Dynamics of Ageing*, eds Banks J, Nazroo J, & Steptoe A (Institute for Fiscal Studies, London), pp 132-161.
7. Taylor R, Conway L, Calderwood L, & Lessof C (2003) Methodology. *Health, Wealth and Lifestyles of the Older Population in England*, eds Marmot M, Banks J, Blundell R, Lessof C, & Nazroo J (Institute for Fiscal Studies, London), pp 357-373.

Table S1 – Associations between SES and covariates

	Wealth quartile Mean (S.D.) or Percent				P
	**1 (highest)**	**2**	**3**	**4 (lowest)**	
Age (yr)	63.63 (7.71)	63.92 (8.22)	64.50 (8.51)	66.26 (9.23)	<0.001
Gender (% women)	52.0%	55.3%	56.7%	62.0%	<0.001
Ethnicity (% White)	98.2%	98.7%	97.6%	96.4%	0.001
Education					
Lower	14.1%	27.1%	41.2%	55.7%	<0.001
Intermediate	38.4%	42.3%	40.2%	32.6%	
Higher	47.5%	30.6%	18.5%	11.7%	
Childhood SES					
Routine	18.8%	29.8%	39.3%	48.0%	<0.001
Intermediate	38.5%	44.7%	44.0%	39.1%	
Managerial/Professional	42.9%	25.6%	16.7%	12.(%	
Long-term conditions (n)					
Prevalence in 2004	0.61 (0.75)	0.71 (0.80)	0.81 (0.88)	1.03 (1.02)	<0.001

Table S2 Outcome variables at baseline and follow-up
Mean (S.D.) or percent

Outcome	Mode*	N	2004/5	2012/13	Difference (95% C.I.)
Physical capability					
Grip strength (Kg)	NV	4003	31.20 (10.97)	27.38 (10.34)	-3.82 (-3.65- -3.99)[1]
Gait speed (m/s)	CAPI	2575	0.95 (0.26)	0.82 (0.26)	-0.12 (-0.11- -0.13)[1]
Chair stand failure %	NV	3440	9.00%	1.9%[3]	
Physical activity index	CAPI	5994	2.31 (1.25)	1.95 (1.37)	-0.36 (-0.32- -0.40)[1]
Sensory function					
Sight impairment %	CAPI	4467	10.9%	15.0%[1]	
Hearing impairment %	CAPI	4465	18.8%	25.5%[1]	
Markers of physiological function					
C-reaction protein (mg/L)	NV	2629	2.73 (2.93)	2.54 (2.94)	-0.19 (-0.06- -0.31)[2]
Fibrinogen (g/L)	NV	2657	3.16 (0.67)	3.00 (0.53)	-0.14 (-0.12- -0.16)[1]
FEV %-predicted	NV	3215	96.09 (23.15)	91.78 (20.47)	-4.30 (-3.66- -4.95)[1]
FVC (L)	NV	3305	3.38 (1.05)	3.18 (0.96)	-0.20 (-0.18- -0.22)[1]
Cognitive function					
Memory (n items)	CAPI	4839	10.72 (3.21)	10.20 (3.80)	-0.52 (-0.42- -0.62)[1]
Verbal fluency (n items)[4]	CAPI	4735	21.05 (6.25)	20.61 (6.77)	-0.44 (-0.27- -0.62)[1]
Processing speed (n items)[4]	CAPI	4413	306.2 (87.9)	295.9 (85.0)	-10.22 (-7.76- -12.69)[1]
Emotional wellbeing					
Enjoyment of life	SC	4087	10.29 (1.65)	9.88 (1.74)	-0.41 (-0.36- -0.48)[1]
Depressive symptoms %	CAPI	4752	19.2%	7.8%[3]	
Social function					
Organizational membership (n)	SC	4018	1.68 (1.47)	1.61 (1.43)	-0.07 (-0.03- -0.11)[1]
Close friends (n)	SC	3487	3.61 (2.21)	3.50 (2.45)	-0.11 (-0.03- -0.20)[2]
Volunteer %	CAPI	4834	24.6%	30.8%	6.25 (4.89- 7.61)[1]
Cultural engagement %	SC	4233	36.9%	33.4%	-3.52 (-1.97- -5.07)[1]

*Mode of assessment CAPI = computer-assisted personal interview; NV = nurse visit; SC = self-completion questionnaire.
[1] P <0.001; [2] P <0.025; [3]Incident (new cases) %; [4]Follow-up assessment in 2010/11

Table S3 Association of SES with 8 year changes in outcomes (standard deviation analyses)

Outcome	Wealth quartile Mean (S.E.) or Odds ratio (95% CI)				P
	1 (highest)	2	3	4 (lowest)	
Physical capability					
Grip strength change	-0.489 (0.029)	-0.600 (0.029)	-0.611 (0.032)	-0.617 (0.039)	0.014
Gait speed change	-0.348 (0.026)	-0.417 (0.026)	-0.508 (0.028)	-0.563 (0.033)	<0.001
Chair stand failure - OR	1 (ref)	2.47 (0.90-6.65)	2.81 (1.03-7.67)	4.20 (1.52-11.62)	0.052
Physical activity index	-0.139 (0.025)	-0.271 (0.025)	-0.351 (0.027)	-0.471 (0.030)	<0.001
Sensory function					
Incident sight impairment - OR	1 (ref)|	1.08 (0.79-1.47)	1.36 (1.00-1.86)	1.60 (1.16-2.20)	0.013
Incident hearing impairment - OR	1 (ref)	1.04 (0.80-1.36)	1.26 (0.96-1.65)	1.30 (0.98-1.74)	0.18
Markers of physiological function					
C-reaction protein change	-0.097 (0.036)	-0.077 (0.035)	-0.005 (0.038)	-0.004 (0.044)	0.064
Fibrinogen change	-0.274 (0.027)	-0.255 (0.026)	-0.152 (0.029)	-0.162 (0.032)	0.002
FEV %-predicted change	-0.126 (0.023)	-0.111 (0.022)	-0.217 (0.025)	-0.300 (0.030)	<0.001
FVC change	-0.167 (0.019)	-0.210 (0.019)	-0.264 (0.021)	-0.314 (0.024)	<0.001
Cognitive function					
Memory change	-0.058 (0.025)	-0.145 (0.025)	-0.190 (0.027)	-0.328 (0.030)	<0.001
Verbal fluency change[1]	-0.036 (0.022)	-0.120 (0.022)	-0.106 (0.023)	-0.152 (0.026)	0.004
Processing speed change[1]	-0.062 (0.022)	-0.126 (0.022)	-0.146 (0.023)	-0.160 (0.027)	0.006
Emotional wellbeing					
Enjoyment of life change	-0.195 (0.027)	-0.195 (0.026)	-0.321 (0.029)	-0.359 (0.034)	<0.001
Incident depressive symptoms – OR	1 (ref)	1.48 (1.00-2.20)	1.65 (1.10-2.48)	2.50 (1.65-2.78)	<0.001
Social function					
Organizational membership change	0.115 (0.023)	-0.025 (0.023)	-0.190 (0.025)	-0.226 (0.030)	<0.001
Close friends change	-0.016 (0.028)	-0.036 (0.028)	-0.040 (0.032)	-0.122 (0.038)	0.039*
Volunteer – OR	1 (ref)	0.92 (0.75-1.13)	0.70 (0.56-0.88)	0.48 (0.37-0.63)	<0.001
Cultural engagement – OR	1 (ref)	0.80 (0.64-0.99)	0.73 (0.58-0.93)	0.51 (0.38-0.67)	<0.001

[1] 6 year change in absolute values
All analyses adjusted for baseline age, age^2, gender, ethnicity, education, childhood SES, number of long-term conditions, and baseline levels of the outcome variable. P for trend across SES groups.
*Not significant after correction for multiple comparisons

**Table S4 Association of SES with 8 year changes in outcomes without baseline level covariates
Standard deviation associations**

Outcome	Wealth quartile Mean (S.E.) or Odds ratio (95% CI)				P
	1 (highest)	2	3	4 (lowest)	
Physical capability					
Grip strength change	-0.533 (0.032)	-0.604 (0.032)	-0.600 (0.035)	-0.540 (0.044)	0.93
Gait speed change	-0.459 (0.031)	-0.431 (0.032)	-0.456 (0.034)	-0.422 (0.039)	0.60
Chair stand failure - OR	1 (ref)	2.47 (0.90-6.65)	2.81 (1.03-7.67)	4.20 (1.52-11.62)	0.052
Physical activity index	-0.237 (0.030)	-0.314 (0.030)	-0.325 (0.032)	-0.303 (0.036)	0.18
Sensory function					
Incident sight impairment - OR	1 (ref)|	1.08 (0.79-1.47)	1.36 (1.00-1.86)	1.60 (1.16-2.20)	0.013
Incident hearing impairment - OR	1 (ref)	1.04 (0.80-1.36)	1.26 (0.96-1.65)	1.30 (0.98-1.74)	0.18
Markers of physiological function					
C-reaction protein change	-0.020 (0.043)	-0.059 (0.042)	-0.071 (0.046)	-0.062 (0.052)	0.54
Fibrinogen change	-0.186 (0.036)	-0.268 (0.034)	-0.222 (0.038)	-0.185 (0.043)	0.80
FEV %-predicted change	-0.189 (0.027)	-0.107 (0.027)	-0.180 (0.029)	-0.246 (0.035)	0.095
FVC change	-0.237 (0.024)	-0.226 (0.024)	-0.218 (0.026)	-0.232 (0.031)	0.87
Cognitive function					
Memory change	-0.114 (0.028)	-0.160 (0.028)	-0.171 (0.030)	-0.247 (0.034)	0.006
Verbal fluency change[1]	-0.085 (0.025)	-0.120 (0.025)	-0.093 (0.026)	-0.100 (0.029)	0.89
Processing speed change[1]	-0.085 (0.026)	-0.134 (0.026)	-0.122 (0.027)	-0.144 (0.032)	0.22
Emotional wellbeing					
Enjoyment of life change	-0.259 (0.030)	-0.220 (0.030)	-0.299 (0.032)	-0.245 (0.038)	0.83
Incident depressive symptoms – OR	1 (ref)	1.48 (1.00-2.20)	1.65 (1.10-2.48)	2.50 (1.65-2.78)	0.001
Social function					
Organizational membership change	0.018 (0.027)	-0.029 (0.027)	-0.136 (0.030)	-0.128 (0.035)	<0.001
Close friends change	-0.058 (0.033)	-0.040 (0.033)	-0.034 (0.037)	-0.045 (0.045)	0.81
Volunteer – OR	1 (ref)	0.92 (0.75-1.13)	0.70 (0.56-0.88)	0.48 (0.37-0.63)	<0.001
Cultural engagement – OR	1 (ref)	0.80 (0.64-0.99)	0.73 (0.58-0.93)	0.51 (0.38-0.67)	<0.001

[1] 6 year SD changes.
All analyses adjusted for baseline age, age^2, gender, ethnicity, education, childhood SES, and number of long-term conditions. P for trend across SES groups.

Table S5 Association of SES with 8 year changes in outcomes - (≤75 years) Standard deviation associations

Outcome	Wealth quartile Mean (S.E.) or Odds ratio (95% CI)				P
	1 (highest)	2	3	4 (lowest)	
Physical capability					
Grip strength change	-0.448 (0.030)	-0.572 (0.030)	-0.578 (0.033)	-0.581 (0.042)	0.019*
Gait speed change	-0.293 (0.028)	-0.379 (0.028)	-0.459 (0.031)	-0.559 (0.037)	<0.001
Chair stand failure - OR	1 (ref)	2.07 (0.74-5.76)	2.13 (0.74-6.18)	2.38 (0.77-7.35)	0.45
Physical activity index	-0.095 (0.027)	-0.201 (0.026)	-0.280 (0.029)	-0.397 (0.034)	<0.001
Sensory function					
Incident sight impairment - OR	1 (ref)	1.05 (0.73-1.50)	1.42 (0.99-2.03)	1.33 (0.90-1.95)	0.15
Incident hearing impairment - OR	1 (ref)	1.00 (0.75-1.34)	1.26 (0.94-1.71)	1.27 (0.92-1.76)	0.23
Markers of physiological function					
C-reaction protein change	-0.152 (0.036)	-0.095 (0.035)	-0.036 (0.039)	0.024 (0.046)	0.003
Fibrinogen change	-0.284 (0.029)	-0.255 (0.027)	-0.152 (0.030)	-0.143 (0.036)	0.001
FEV %-predicted change	-0.161 (0.023)	-0.142 (0.023)	-0.250 (0.025)	-0.303 (0.031)	<0.001
FVC change	-0.156 (0.019)	-0.194 (0.019)	-0.255 (0.022)	-0.292 (0.026)	<0.001
Cognitive function					
Memory change	-0.004 (0.026)	-0.068 (0.026)	-0.130 (0.028)	-0.273 (0.033)	<0.001
Verbal fluency change[1]	-0.005 (0.024)	-0.172 (0.024)	-0.063 (0.025)	-0.093 (0.029)	0.041*
Processing speed change[1]	-0.045 (0.023)	-0.110 (0.023)	-0.149 (0.024)	-0.127 (0.029)	0.030*
Emotional wellbeing					
Enjoyment of life change	-0.162 (0.028)	-0.167 (0.028)	-0.271 (0.030)	-0.355 (0.037)	<0.001
Incident depressive symptoms – OR	1 (ref)	1.47 (0.95-2.28)	1.74 (1.11-2.75)	2.83 (1.78-4.50)	<0.001
Social function					
Organizational membership change	0.124 (0.024)	0.007 (0.024)	-0.155 (0.026)	-0.199 (0.032)	<0.001
Close friends change	0.001 (0.029)	-0.013 (0.029)	-0.030 (0.033)	-0.121 (0.041)	0.021*
Volunteer – OR	1 (ref)	1.01 (0.81-1.26)	0.78 (0.61-0.99)	0.50 (0.37-0.66)	<0.001
Cultural engagement – OR	1 (ref)	0.78 (0.62-0.98)	0.73 (0.57-0.93)	0.48 (0.36-0.65)	<0.001

[1] 6 year SD changes.
All analyses adjusted for baseline age, age^2, gender, ethnicity, education, childhood SES, number of long-term conditions, and baseline levels of the outcome variable.
P for trend across SES groups.
*Not significant after correction for multiple comparisons

Table S6 Association of SES with 8 year changes in outcomes, additionally controlling for smoking; Standard deviation associations

**Outcome**	**Wealth quartile Mean (S.E.) or Odds ratio (95% CI)**				**P**
	**1 (highest)**	**2**	**3**	**4 (lowest)**	
Physical capability					
Grip strength change	-0.493 (0.029)	-0.601 (0.029)	-0.611 (0.032)	-0.612 (0.039)	0.024*
Gait speed change	-0.358 (0.026)	-0.422 (0.026)	-0.507 (0.028)	-0.552 (0.033)	0.001
Chair stand failure - OR	1 (ref)	2.47 (0.92-6.64)	2.79 (1.02-7.60)	4.12 (1.48-11.46)	0.059
Physical activity index	-0.148 (0.025)	-0.278 (0.025)	-0.351 (0.026)	-0.447 (0.030)	<0.001
Sensory function					
Incident sight impairment - OR	1 (ref)	1.07 (0.78-1.46)	1.33 (0.97-1.81)	1.48 (1.08-2.05)	0.055
Incident hearing impairment - OR	1 (ref)	1.04 (0.80-1.36)	1.26 (0.96-1.65)	1.32 (0.99-1.77)	0.16
Markers of physiological function					
C-reaction protein change	-0.094 (0.036)	-0.076 (0.035)	-0.005 (0.038)	-0.009 (0.044)	0.086
Fibrinogen change	-0.269 (0.027)	-0.251 (0.026)	-0.150 (0.029)	-0.174 (0.033)	0.007
FEV %-predicted change	-0.137 (0.022)	-0.114 (0.022)	-0.218 (0.024)	-0.271 (0.029)	<0.001
FVC change	-0.173 (0.019)	-0.213 (0.019)	-0.264 (0.020)	-0.301 (0.024)	<0.001
Cognitive function					
Memory change	-0.068 (0.025)	-0.154 (0.025)	-0.189 (0.026)	-0.307 (0.031)	<0.001
Verbal fluency change[1]	-0.043 (0.023)	-0.124 (0.022)	-0.106 (0.023)	-0.139 (0.026)	0.019*
Processing speed change[1]	-0.067 (0.022)	-0.128 (0.022)	-0.145 (0.023)	-0.154 (0.027)	0.016
Emotional wellbeing					
Enjoyment of life change	-0.201 (0.027)	-0.199 (0.026)	-0.320 (0.029)	-0.348 (0.034)	<0.001
Incident depressive symptoms – OR	1 (ref)	1.49 (1.00-2.21)	1.59 (1.06-2.40)	2.27 (1.50-3.44)	0.002
Social function					
Organizational membership change	0.109 (0.023)	-0.028 (0.023)	-0.189 (0.025)	-0.216 (0.030)	<0.001
Close friends change	-0.021 (0.028)	-0.039 (0.028)	-0.038 (0.032)	-0.112 (0.038)	0.083
Volunteer – OR	1 (ref)	1.00 (0.81-1.24)	0.77 (0.61-0.98)	0.56 (0.42-0.74)	<0.001
Cultural engagement – OR	1 (ref)	0.80 (0.64-0.99)	0.74 (0.59-0.94)	0.53 (0.40-0.71)	<0.001

[1] 6 year SD changes.
All analyses adjusted for baseline age, age^2, gender, ethnicity, education, childhood SES, number of long-term conditions, and baseline levels of the outcome variable.
P for trend across SES groups. *Not significant after correction for multiple comparisons
